# Supplementary material for: Rebalancing of actomyosin contractility enables mammary tumor formation upon loss of E-cadherin
Source: Nat Commun. 2019 Aug 23;10:3800. doi: 10.1038/s41467-019-11716-6 (PMC6707221; doi:10.1038/s41467-019-11716-6)
Supplement: Supplementary file 1 — Supplementary Information [file 41467_2019_11716_MOESM1_ESM.pdf]

Rebalancing of actomyosin contractility enables mammary tumor formation upon loss of E-cadherin

Schipper et al.

Supplementary information

Supplementary Figure 1

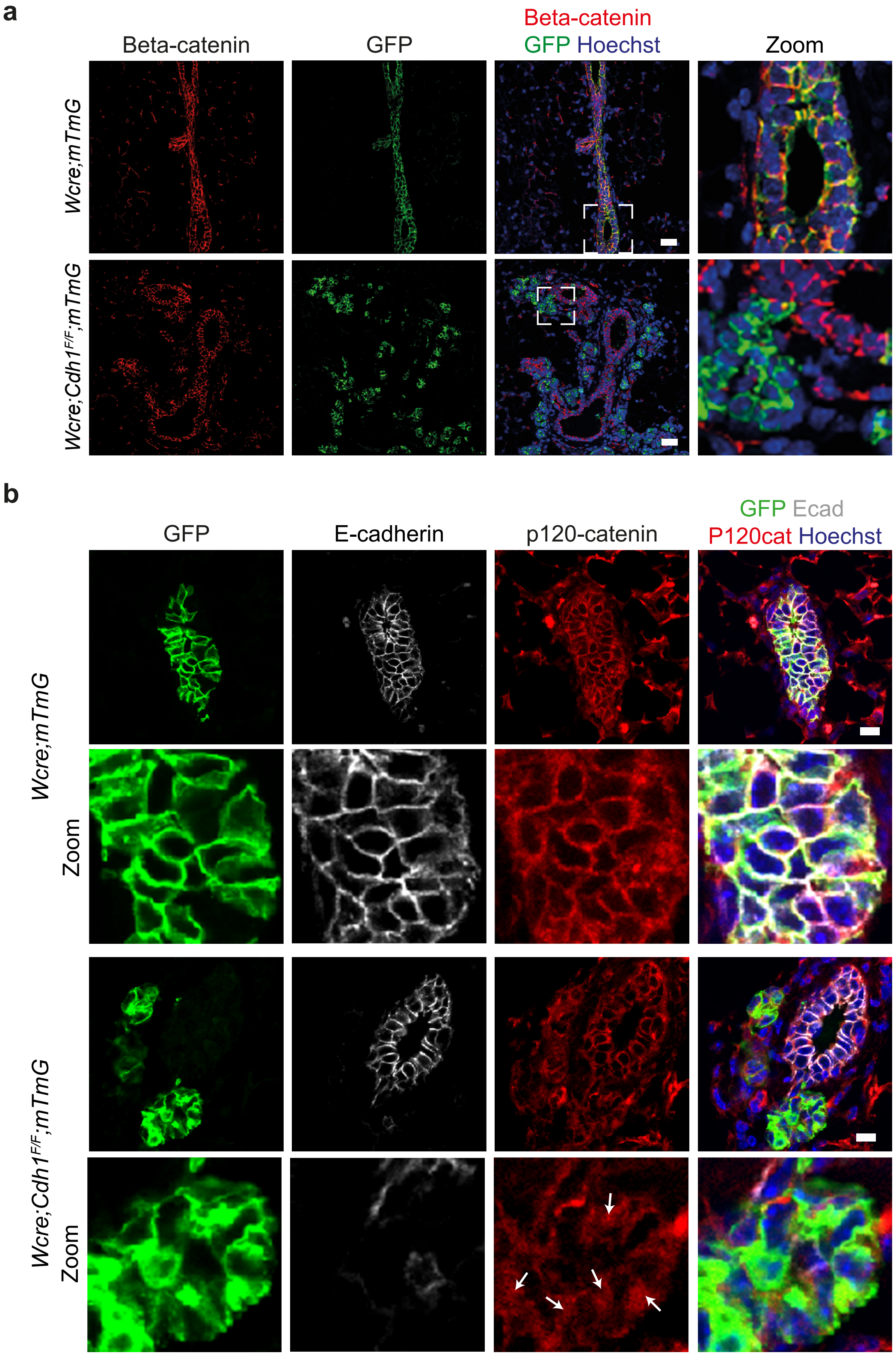

Supplementary Figure 1 Immunofluorescence analysis of Beta-catenin and P120 catenin (**a**) IF analysis of *Wcre;mTmG* and *Wcre;Cdh1*$^{F/F}$;*mTmG* female mice stained for beta-catenin, GFP and Hoechst. Scale bar is 40 μM. (**b**) IF analysis of *Wcre;mTmG* and *Wcre;Cdh1*$^{F/F}$;*mTmG* female mice stained for p120-catenin, GFP, E-cadherin and Hoechst. Scale bar is 20 μM.

Supplementary Figure 2

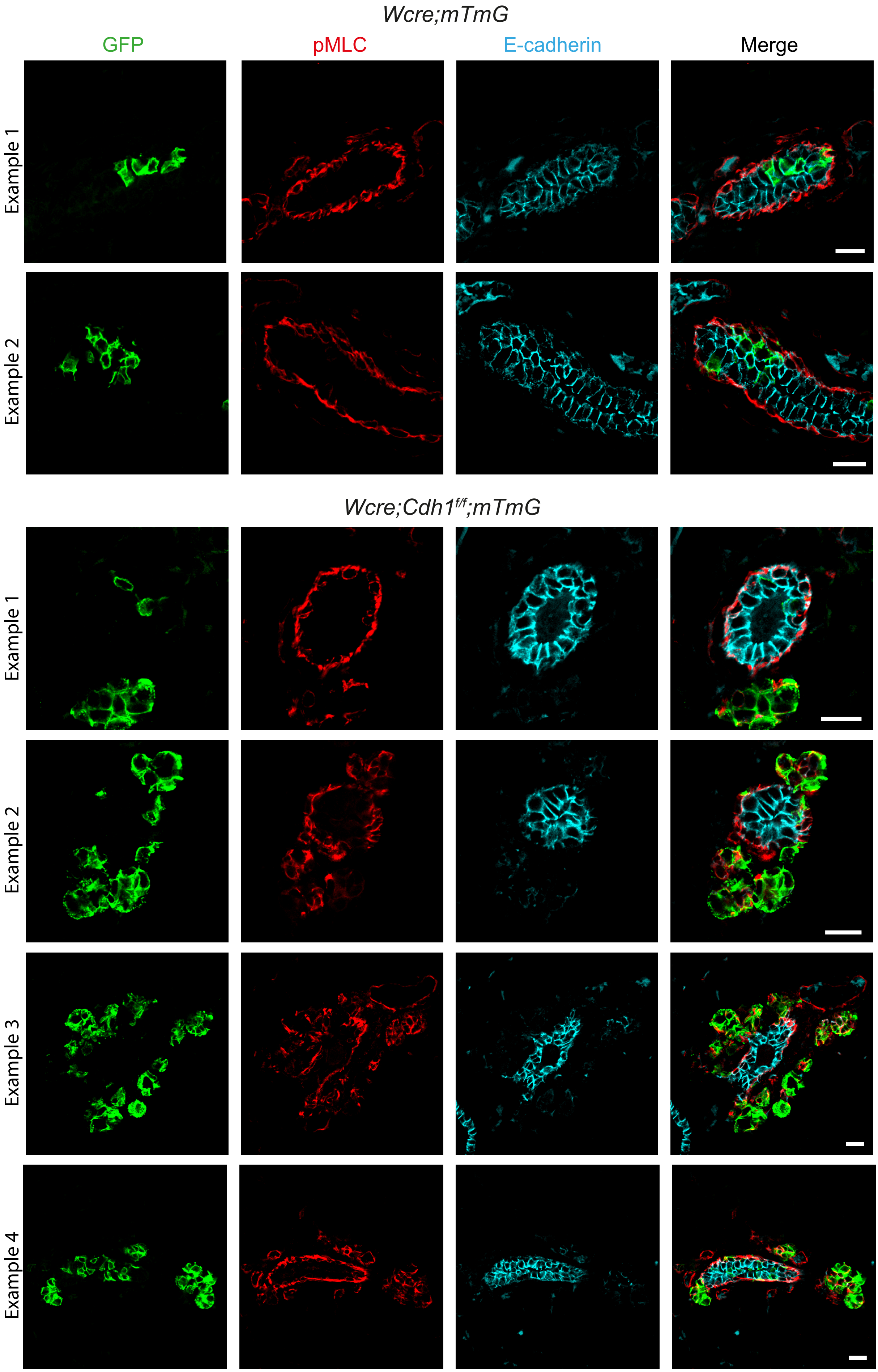

Supplementary Figure 2 E-cadherin loss increases actomyosin contractility IF analysis of *Wcre;mTmG* and *Wcre;Cdh1*$^{F/F}$*;mTmG* female mice stained for pMLC, GFP and Hoechst. Scale bar is 20μM.

Supplementary Figure 3

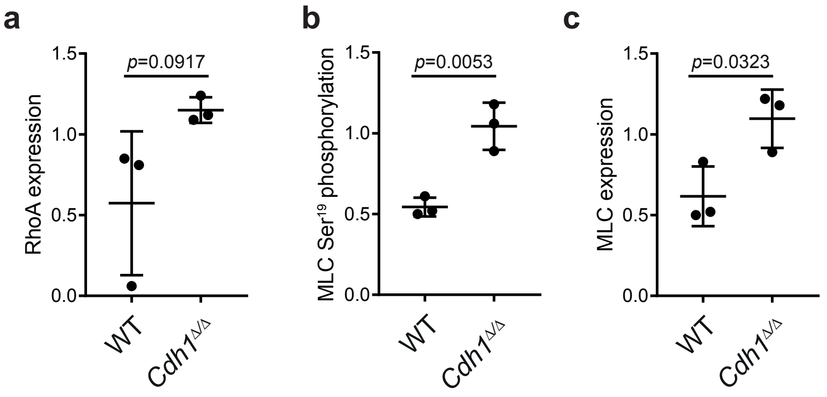

Supplementary Figure 3 E-cadherin deficient MMECs have increased RhoA expression and myosin light chain activity (**a-c**) Quantification of immunoblots of RhoA (**a**), pMLC (**b**) and MLC (**c**) by densitometry normalized to the actin loading control. Data are mean ± standard deviation of three independent experiments. *p*-values were calculated using an unpaired two tailed t-test. Source data are provided as a Source Data file.

Supplementary Figure 4

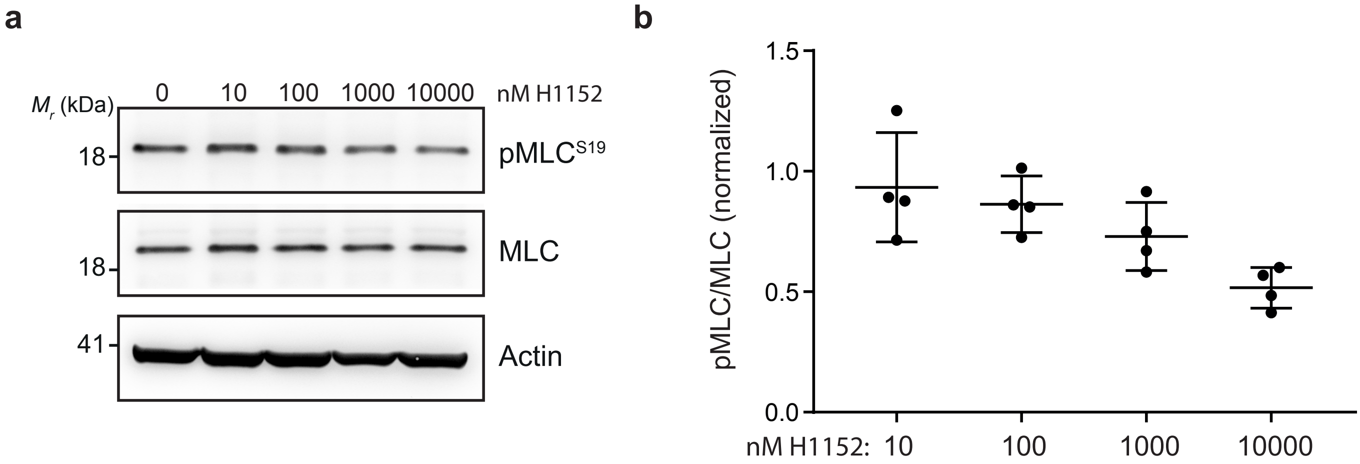

Supplementary Figure 4 Optimal ROCK inhibition only partially inhibits MLC phosphorylation (**a**) Western blot analysis and Quantification of E-cadherin deficient MMECs exposed to various concentrations of H1152 for 1 hour post seeding stained for pMLC Ser19, MLC and actin. (**b**) Quantification of the amount of MLC phosphorylation. Ratios were normalized to Vehicle (0 nM H1152) for each replicate. Data are mean ± standard deviation of four independent experiments. Source data are provided as a Source Data file.

Supplementary Figure 5

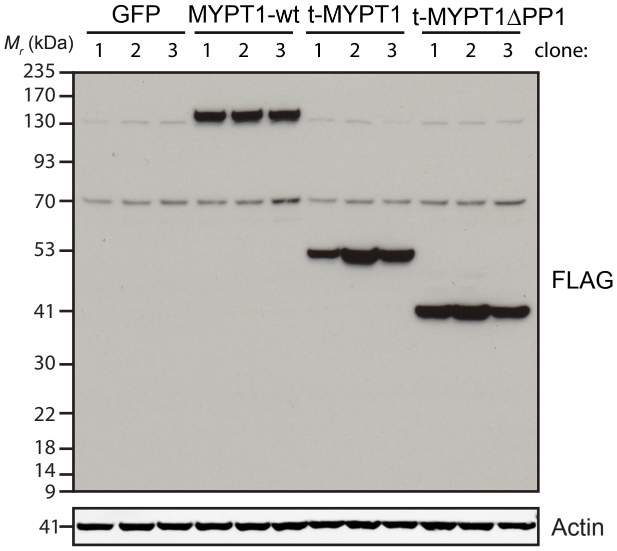

Supplementary Figure 5 Expression of MYPT1 variants in E-cadherin-deficient MMECs. Western blot analysis of FLAG tagged MYPT1-wt, t-MYPT1 and t-MYPT1ΔPP1 protein expression. β-actin was used as a loading control.

Supplementary Figure 6

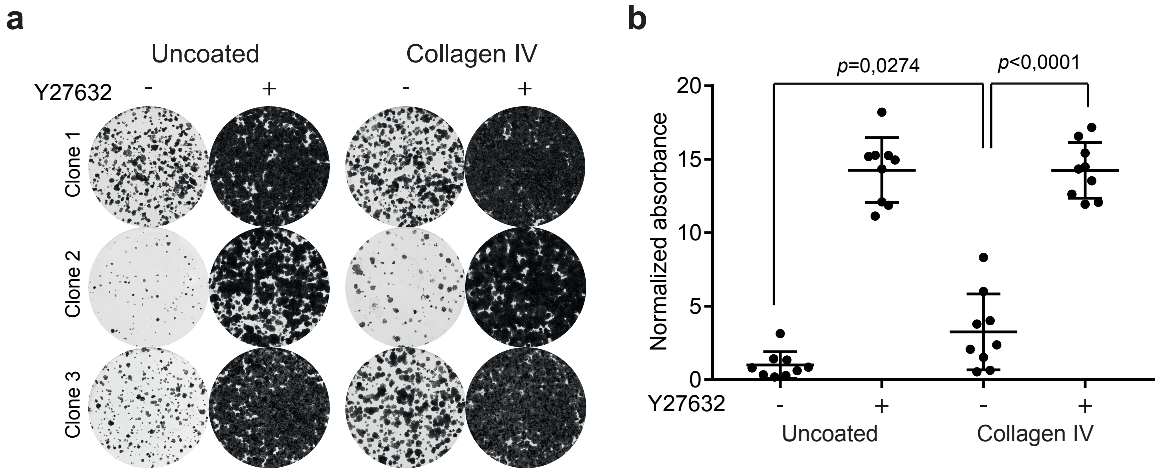

Supplementary Figure 6 Collagen IV slightly increases survival of E-cadherin deficient MMECs. Representative images (**a**) and Quantification (**b**) of clonogenic assays with *Wcre;Cdh-1*$^{F/F}$*;mTmG* MMECs seeded on collagen IV-coated wells. Data are mean ± standard deviation of three independent experiments with 3 clones per experiment. *p*-values were calculated using an unpaired two tailed t-test. Source data are provided as a Source Data file.

Supplementary Figure 7

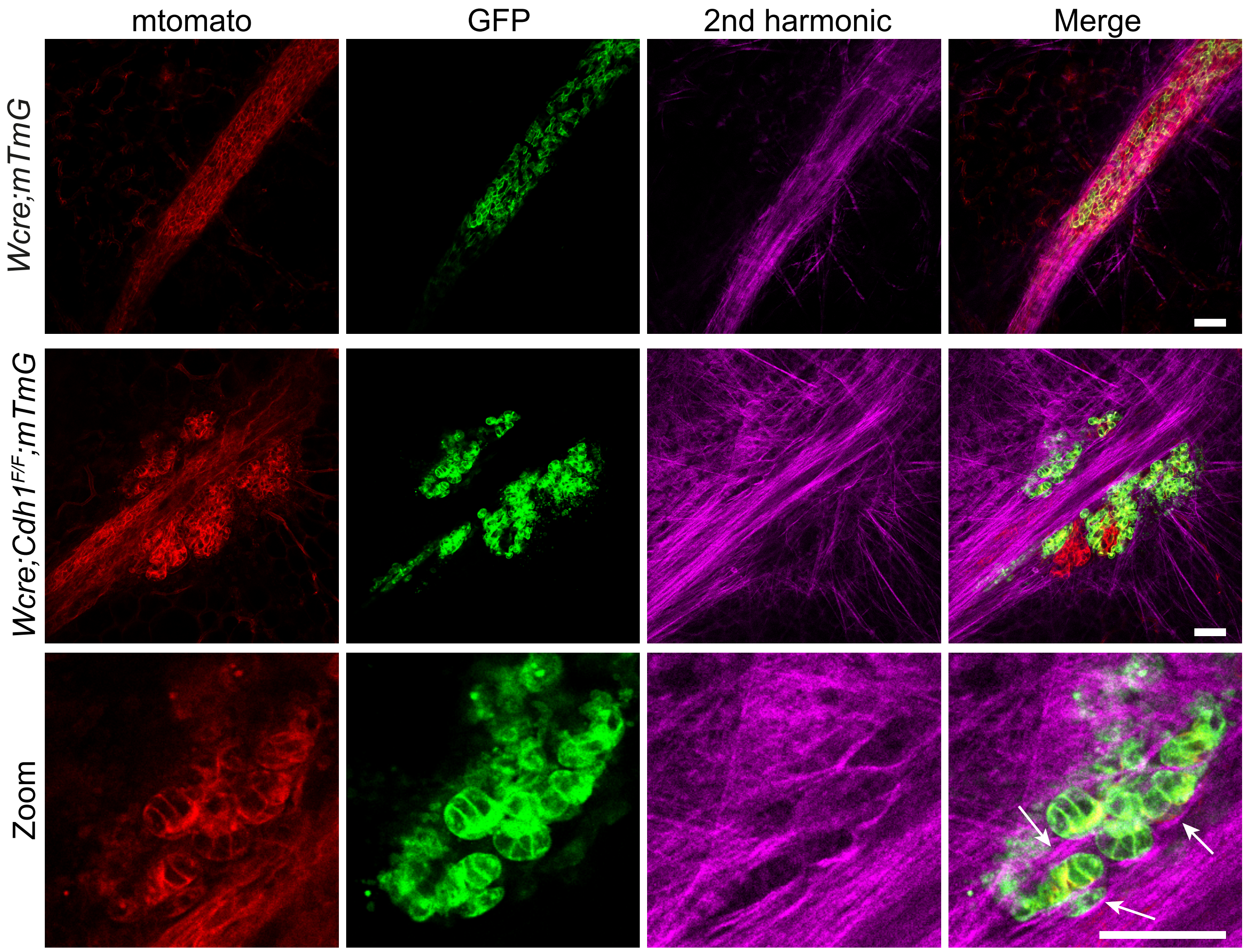

Supplementary Figure 7 Clusters of extruded E-cadherin deficient cells are surrounded by fibrillar collagen Still images derived from *in vivo* intravital imaging of the mammary gland of 8-week-old *Wcre;mTmG* and *Wcre;Cdh1$^{F/F}$;mTmG* mice. GFP signal is visualized in green, mTomato in red and 2nd harmonic generation used to detect fibrillar collagen is depicted as magenta. Scale bar is 50 μM.

Supplementary Figure 8

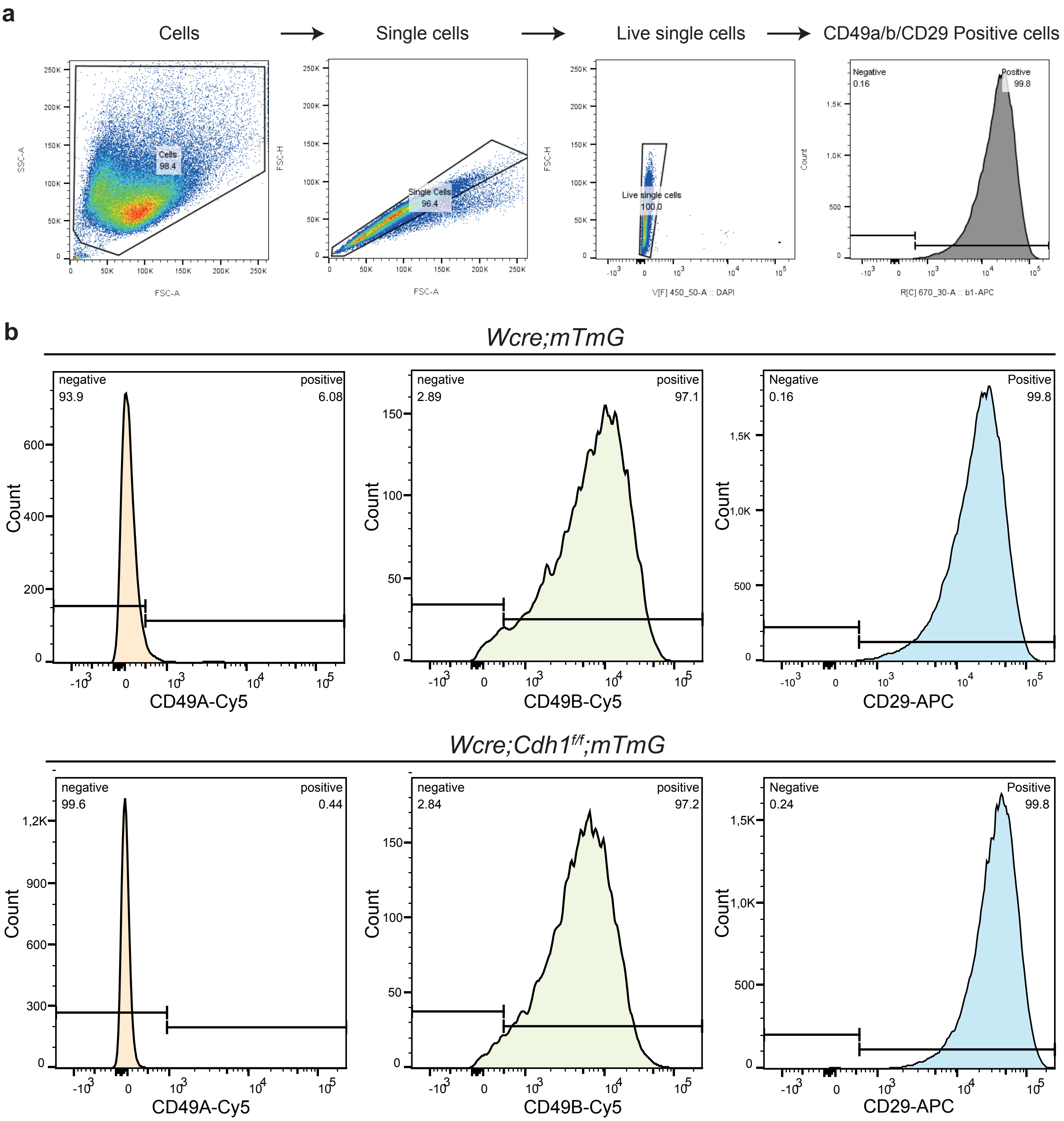

Supplementary Figure 8 Collagen Integrin expression of MMECs (**a**) Schematic overview of the gating strategy**.** (**b**) FACS analysis of *Wcre;mTmG* and *Wcre;Cdh1$^{F/F}$;mTmG* derived MMECs stained for integrin alpha 1 (CD49a),integrin alpha 2 (CD49b) and integrin beta-1 (CD29).

Supplementary Figure 9

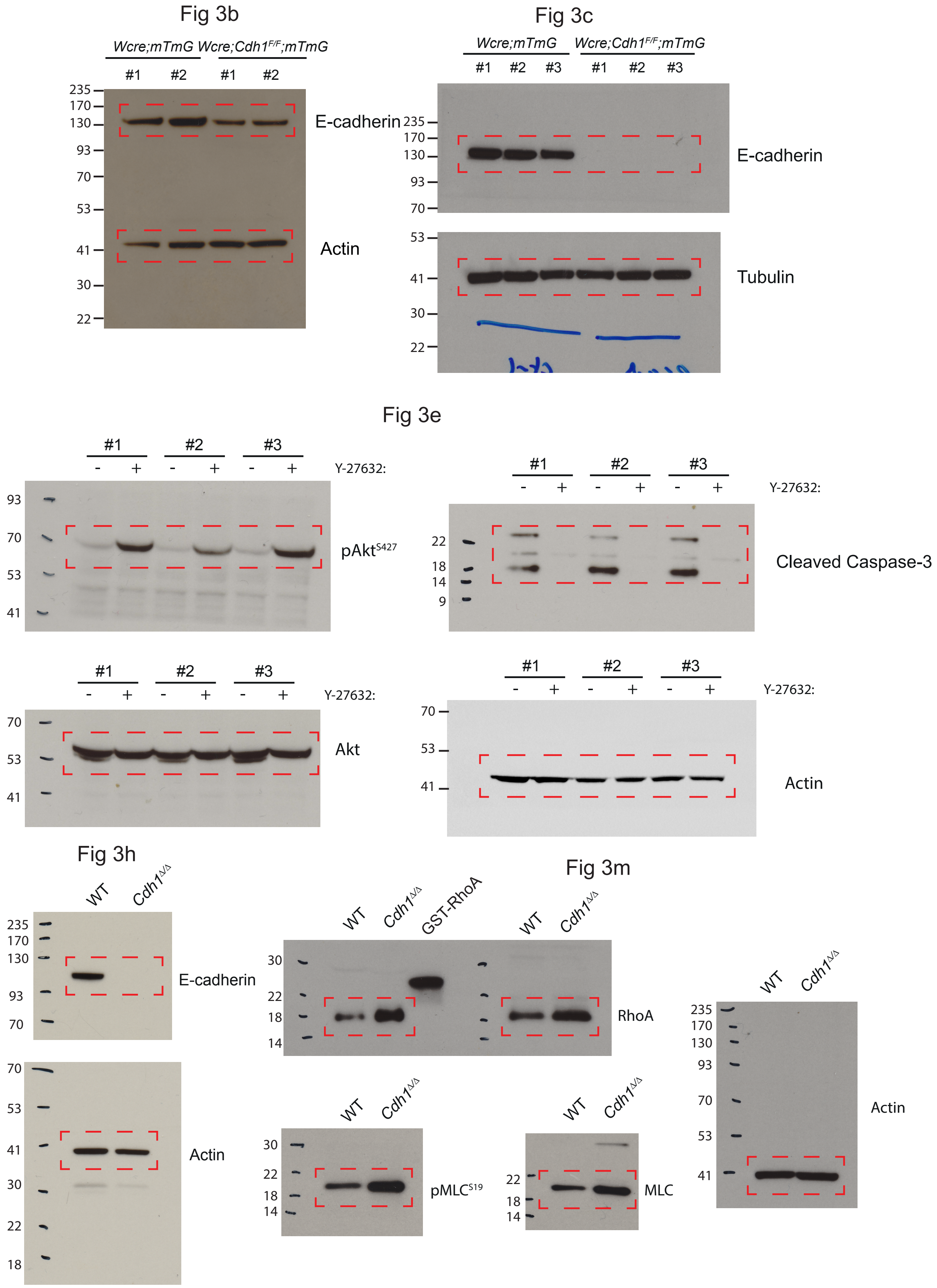

Supplementary Figure 9

Fig 5b

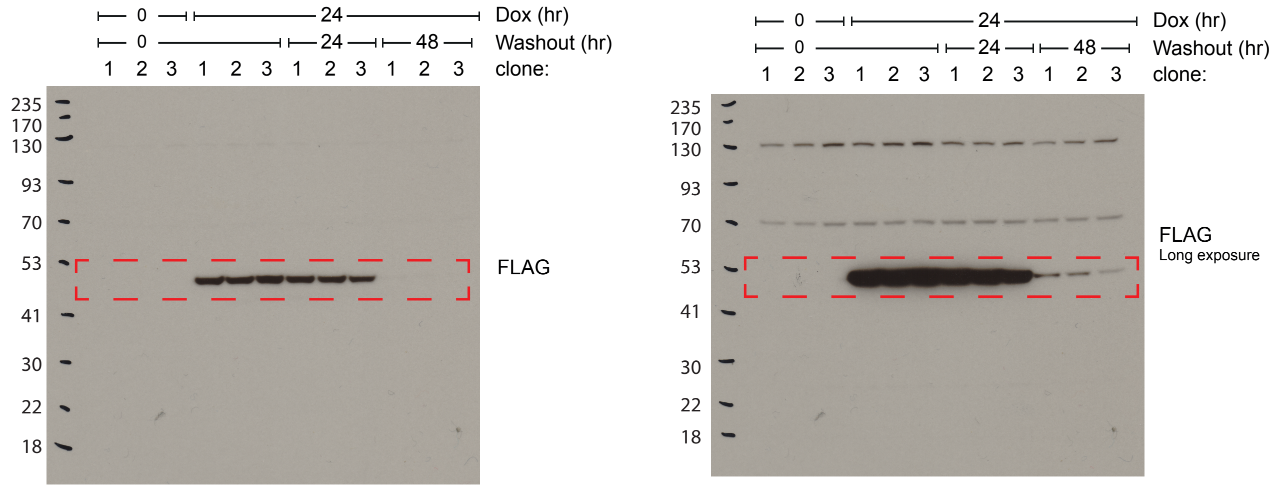

Fig 5b

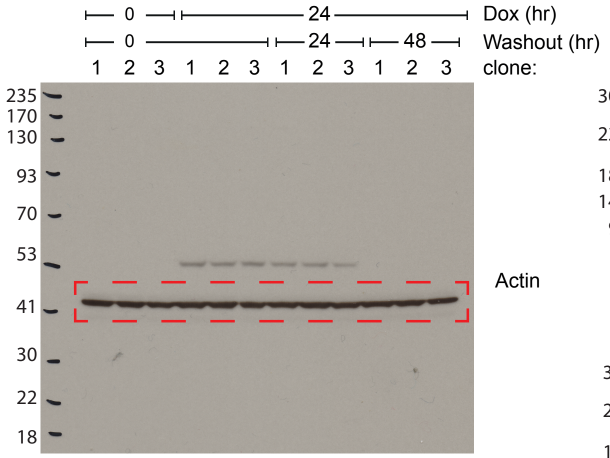

Fig 6h

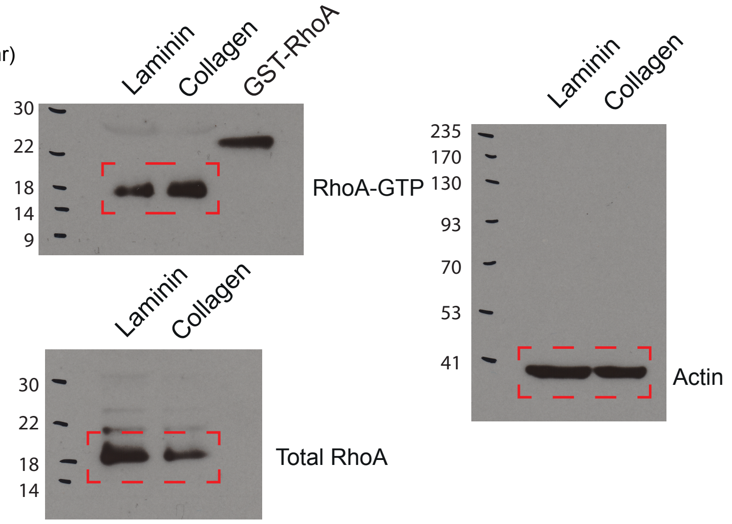

Supplementary Figure 9

Fig s3

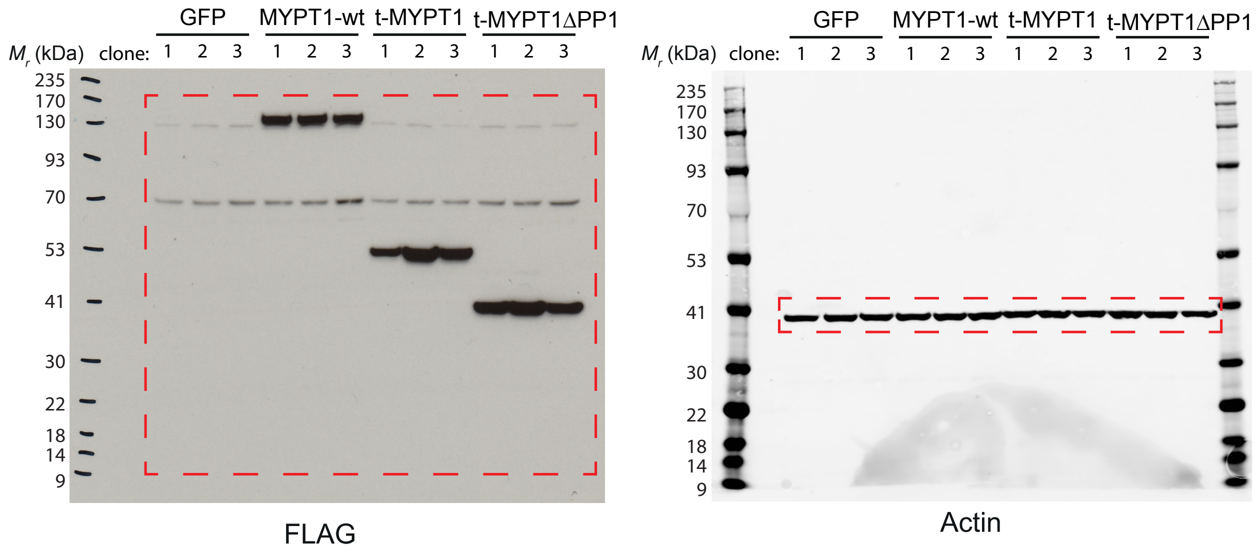

Fig s4

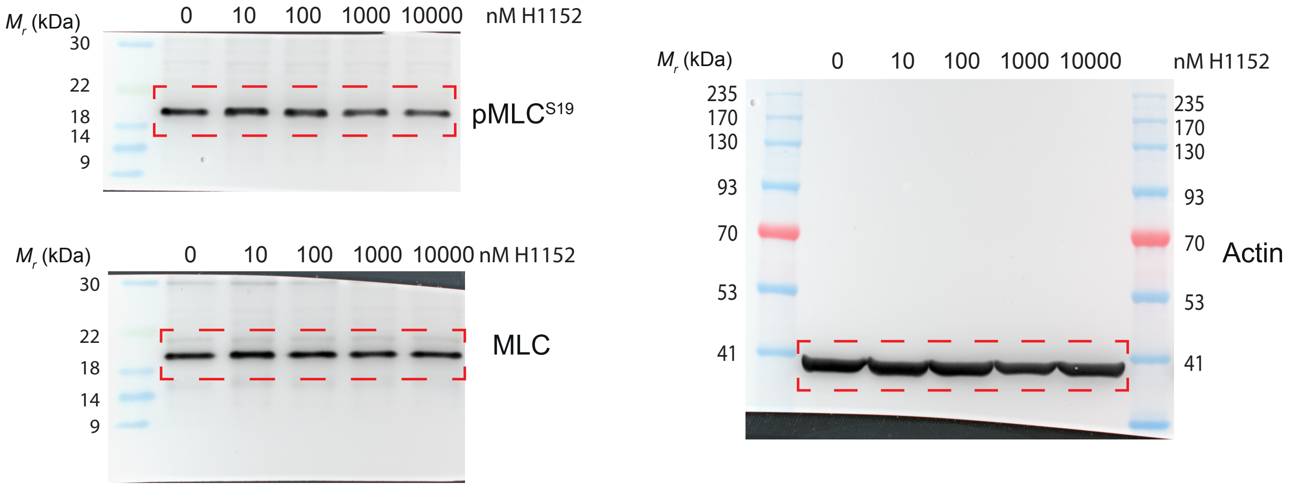

Supplementary Table 1

List of used primers

Information	Experiment		Sequence
MYPT1 in SIN.LV.SF-T2A-puro and SIN.LV.SF	Addition AgeI -AgeI overhangs	FWD	GTCACCACCGGTATGAAGATGGCGGACGCGAAG
MYPT1 in SIN.LV.SF-T2A-puro	Addition Age1-AgeI overhangs + FLAG tag	REV	CGACTCACCGGTCTTGTCGTCATCGTCTTTGTAGTCCTTGGAAAGTTTGCTTATAAC
MYPT1 in SIN.LV.SF	Addition AgeI -AgeI overhangs	REV	CGACTCGTCGACTCAGATATTCTTCTTTCTAGAGCTC
tMYPT1 in SIN.LV.SF-T2A-puro	Addition BamHI-AgeI overhangs	FWD	GTCACCGGATCCATGAAGATGGCGGACGCGAAG
tMYPT1 and MYPT1ΔPP1 in SIN.LV.SF-T2A-puro	Addition BamHI-AgeI overhangs	REV	CGACTCACCGGTCTTGTCGTCATCGTCTTTGTAG
MYPT1ΔPP1 in SIN.LV.SF and SIN.LV.SF-T2A-puro	Addition BamHI-SalI/BamHI-AgeI overhangs and start codon	FWD	GTACCGGATCCATGCTGGTAGAAAATGGAGCAAATATC
tMYPT1 in SIN.LV.SF	Addition AgeISal1 overhangs	FWD	ACCGGTATGAAGATGGCGGACG
tMYPT1 and MYPT1ΔPP1 in SIN.LV.SF	Addition AgeI/BamHI-Sal1 overhangs	REV	GTCGACTCACTTGTCGTCATCGTC
pINDUCER20 cassete into SIN.LV.SF	Addition Nhe1 Sal1 overhangs	FWD	GTACCGCTAGCTTTACCACTCCCTATCAGTG
pINDUCER20 cassete into SIN.LV.SF	Addition Nhe1 Sal1 overhangs	REV	CGACTCGTCGACTTACCCGGGGAGCATGTCAAG
