# Supplementary material for: Rebalancing of actomyosin contractility enables mammary tumor formation upon loss of E-cadherin
Source: Nat Commun. 2019 Aug 23;10:3800. doi: 10.1038/s41467-019-11716-6 (PMC6707221; doi:10.1038/s41467-019-11716-6)
Supplement: Supplementary file 3 — Description of Additional Supplementary Files [file 41467_2019_11716_MOESM3_ESM.pdf]

Description of Additional Supplementary Files

File Name: Supplementary Movie 1
Description: Representative example of Intravital imaging of a mammary duct from a *Wcre;Cdh1$^{F/F}$;mTmG* female mouse.

File Name: Supplementary Movie 2
Description: Representative example of Intravital imaging of a mammary duct from a *Wcre;mTmG* female mouse.

File Name: Supplementary Movie 3
Description: Zoom of a mammary duct from a Wcre;Cdh1$^{F/F}$;mTmG female mice depicting membrane blebbing.

File Name: Supplementary Movie 4
Description: Time laps imaging of merged bright field and GFP fluorescence of *Wcre;Cdh1$^{F/F}$;mTmG* MMECs *in vitro*.
