# Supplementary figures and images for: Rebalancing of actomyosin contractility enables mammary tumor formation upon loss of E-cadherin
Source: Nat Commun. 2019 Aug 23;10:3800. doi: 10.1038/s41467-019-11716-6 (PMC6707221; doi:10.1038/s41467-019-11716-6)

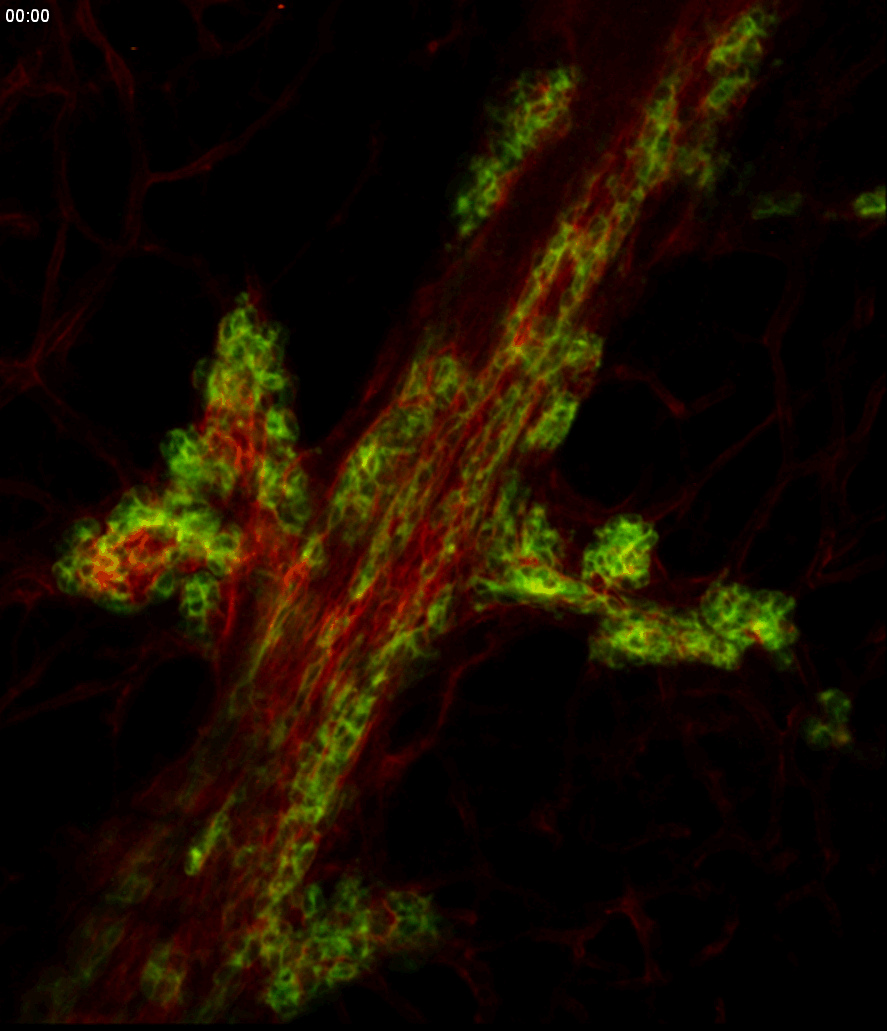

Supplement: Supplementary file 4 — Supplementary Movie 1 [file 41467_2019_11716_MOESM4_ESM.gif]

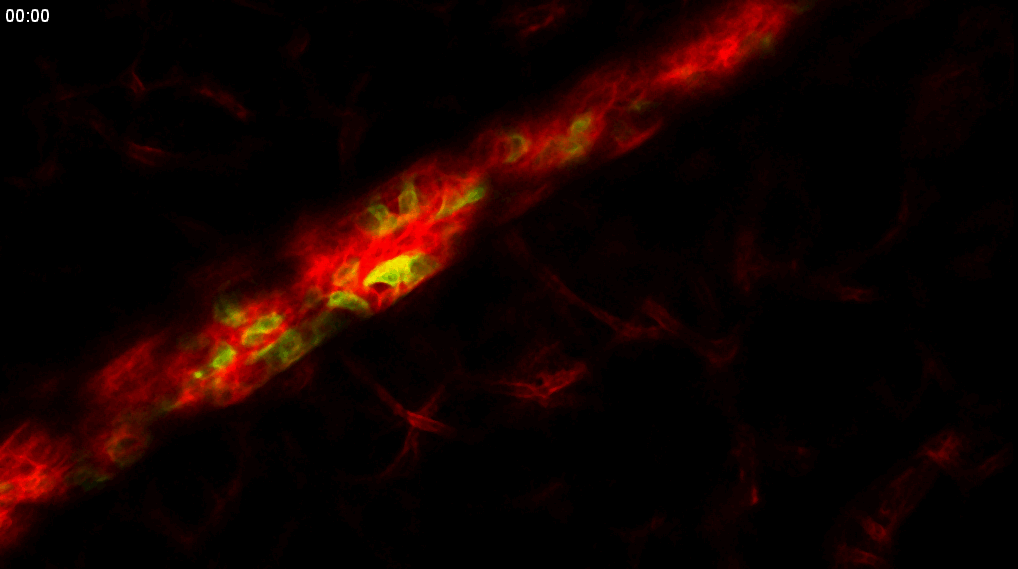

Supplement: Supplementary file 5 — Supplementary Movie 2 [file 41467_2019_11716_MOESM5_ESM.gif]

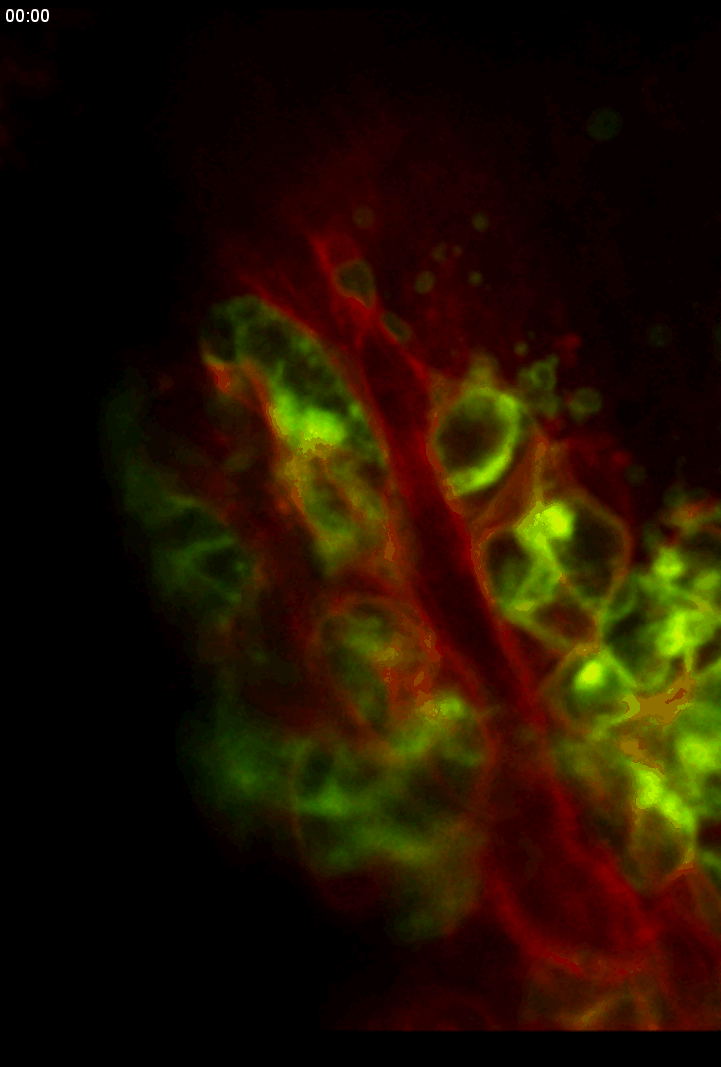

Supplement: Supplementary file 6 — Supplementary Movie 3 [file 41467_2019_11716_MOESM6_ESM.gif]
