# Supplementary material for: Rebalancing of actomyosin contractility enables mammary tumor formation upon loss of E-cadherin
Source: Nat Commun. 2019 Aug 23;10:3800. doi: 10.1038/s41467-019-11716-6 (PMC6707221; doi:10.1038/s41467-019-11716-6)
Supplement: Supplementary file 8 — Reporting Summary [file 41467_2019_11716_MOESM8_ESM.pdf]

nature research

Corresponding author(s): Micha Nethe and Jos Jonkers

Last updated by author(s): Jul 16, 2019

Reporting Summary

Nature Research wishes to improve the reproducibility of the work that we publish. This form provides structure for consistency and transparency in reporting. For further information on Nature Research policies, see Authors & Referees and the Editorial Policy Checklist.

Statistics

For all statistical analyses, confirm that the following items are present in the figure legend, table legend, main text, or Methods section.

n/a	Confirmed	
☐	☒	The exact sample size (*n*) for each experimental group/condition, given as a discrete number and unit of measurement
☐	☒	A statement on whether measurements were taken from distinct samples or whether the same sample was measured repeatedly
☐	☒	The statistical test(s) used AND whether they are one- or two-sided<br>*Only common tests should be described solely by name; describe more complex techniques in the Methods section.*
☒	☐	A description of all covariates tested
☐	☒	A description of any assumptions or corrections, such as tests of normality and adjustment for multiple comparisons
☐	☒	A full description of the statistical parameters including central tendency (e.g. means) or other basic estimates (e.g. regression coefficient) AND variation (e.g. standard deviation) or associated estimates of uncertainty (e.g. confidence intervals)
☐	☒	For null hypothesis testing, the test statistic (e.g. *F*, *t*, *r*) with confidence intervals, effect sizes, degrees of freedom and *P* value noted<br>*Give P values as exact values whenever suitable.*
☒	☐	For Bayesian analysis, information on the choice of priors and Markov chain Monte Carlo settings
☒	☐	For hierarchical and complex designs, identification of the appropriate level for tests and full reporting of outcomes
☒	☐	Estimates of effect sizes (e.g. Cohen's *d*, Pearson's *r*), indicating how they were calculated

Our web collection on statistics for biologists contains articles on many of the points above.

Software and code

Policy information about availability of computer code

Data collection	Leica Acquisition Suite (LAS) AF and X, Zeis ZEN v2.3, Gelcount software v1.1.2.0, Odyssey Application software version 3.0.16
Data analysis	Graphpad Prism 7.03, ImageJ(FIJI), Imagescope v12.0.0, Adobe creative suite, Microsoft Excell, Odyssey Application software version 3.0.16

For manuscripts utilizing custom algorithms or software that are central to the research but not yet described in published literature, software must be made available to editors/reviewers. We strongly encourage code deposition in a community repository (e.g. GitHub). See the Nature Research guidelines for submitting code & software for further information.

Data

Policy information about availability of data

All manuscripts must include a data availability statement. This statement should provide the following information, where applicable:

- Accession codes, unique identifiers, or web links for publicly available datasets
- A list of figures that have associated raw data
- A description of any restrictions on data availability

Source data for figures 1-7 and supplementary figures 3-6 can be found in supplementary table 3. All other data supporting the findings of this study can be obtained from the corresponding authors upon reasonable request.

Field-specific reporting

Please select the one below that is the best fit for your research. If you are not sure, read the appropriate sections before making your selection.

☒ Life sciences ☐ Behavioural & social sciences ☐ Ecological, evolutionary & environmental sciences

For a reference copy of the document with all sections, see nature.com/documents/nr-reporting-summary-flat.pdf

Life sciences study design

All studies must disclose on these points even when the disclosure is negative.

Sample size	No sample size calculation was performed. Sample sizes were chosen based on experience with previous experiments
Data exclusions	No samples were excluded from analysis
Replication	All attempts at replication were successful
Randomization	Cells and mice were randomly allocated to the various conditions.
Blinding	Investigators were not blinded to allocation of mice to groups

Reporting for specific materials, systems and methods

We require information from authors about some types of materials, experimental systems and methods used in many studies. Here, indicate whether each material, system or method listed is relevant to your study. If you are not sure if a list item applies to your research, read the appropriate section before selecting a response.

Materials & experimental systems

n/a	Involved in the study	
☐	☒	Antibodies
☐	☒	Eukaryotic cell lines
☒	☐	Palaeontology
☐	☒	Animals and other organisms
☒	☐	Human research participants
☒	☐	Clinical data

Methods

n/a	Involved in the study	
☒	☐	ChIP-seq
☐	☒	Flow cytometry
☒	☐	MRI-based neuroimaging

Antibodies

Antibodies used

Antibody Application Dilution Species Supplier catalog nr Clone lot
GFP IF 1:200 Chicken Abcam ab13970 polyclonal GR89472-19
Cytokeratin 8 IF/IHC 1:200/1:600 Rat DSHB TROMA-1 TROMA-1 -
Cytokeratin 14 IF 1:200 Rabbit Covance PRB-155P polyclonal D141F01918
phospho MLC (Ser20) IF 1:100 Rabbit Cell Signaling 3671 -
E-cadherin IF 1:200 Mouse E-bioscience 610181 36/E-Cadherin 7187865
Laminin IF 1:100 Rabbit Abcam ab11575 Polyclonal GR68714-12
anti-Rat Alexa Fluor 647 IF 1:1000 Goat Invitrogen A21247 polyclonal 1453307
anti Rabbit Alexa Fluor 568 IF 1:1000 Goat Invitrogen A11011 polyclonal 1246464
anti-Rat Alexa Fluor 568 IF 1:1000 Goat Invitrogen 11077 polyclonal 1512105
anti-mouse-Alexa Fluor 488 IF 1:1000 Goat Invitrogen A21141 polyclonal 1298479
anti-Mouse Alexa Fluor 405 IF 1:100 Goat Invitrogen A31553 polyclonal 1512096
Anti Chicken Alexa Fluor 488 IF 1:1000 Goat Invitrogen A-11039 polyclonal 1458638
E-cadherin WB 1:1000 Rat Invitrogen #13-1900 ECCD-2 -
Cleaved caspase-3 WB 1:1000 Rabbit Cell Signaling 9661 - 43
AKT1 WB 1:1000 Rabbit Cell Signaling 2938 - 4
phospho-AKT(Ser473) WB 1:1000 Rabbit Cell Signaling 4060 - 14
FLAG WB 1:1000 Rabbit Sigma F7425 polyclonal 086M4803V
MLC WB 1:1000 Rabbit Cell Signaling 3672 - 6
phospho MLC (Ser20) WB 1:1000 Rabbit Abcam ab2480 polyclonal GR3189077-1
Tubulin WB 1:1000 Mouse Sigma T9026 DM1A 092M4792
β-actin WB 1:20000 Mouse Sigma A5441 AC-15 102M4804V
RhoA (part of kit) WB 1:500 Mouse Cytoskeleton BK036 - 49
Anti-Rabbit HRP WB 1:2500 Goat DAKO P0260 Polyclonal 20045584
Anti-Mouse HRP WB 1:2500 Rabbit DAKO P02448 Polyclonal 20053537
Anti Rat HRP WB 1:2500 Goat Invitrogen 62-9520 Polyclonal SB241986

	Anti-mouse IRDye 680nm WB 1:5000 Donkey Li-COR 926-32222 Polyclonal C70419-08<br>GFP IHC 1:1000 Rabbit Abcam ab6556 Polyclonal GR3216572-1<br>alpha1 Integrin (CD49a) FC 1:100 Armenian Hamster BD biosciences 555001 Ha31/8 M005383<br>alpha2 Integrin (CD49b) FC 1:100 Armenian Hamster Thermo Fischer 14-0491-82 HMa2 61439<br>Cy5-Goat anti Hamster FC 1:400 Goat Jackson laboratories Polyclonal<br>Beta1 integrin-APC (CD29) FC 1:50 Armenian hamster Thermo Fischer 14-0291-82 Monoclonal 4289643
Validation	Commercially available antibodies were validated by the manufacturer and all of them are have been previously used in multiple publications. For further details including lists of publications please refer to the suppliers websites.

Eukaryotic cell lines

Policy information about cell lines

Cell line source(s)	All MMEC lines were generated in house
Authentication	None of the cell lines were authenicated
Mycoplasma contamination	All cell lines were tested negative for Mycoplasma contamination
Commonly misidentified lines (See ICLAC register)	No commonly misidentified cell lines were used

Animals and other organisms

Policy information about studies involving animals; ARRIVE guidelines recommended for reporting animal research

Laboratory animals	The investigators used in house generated genetically modified female FVB mice of various ages as indicated in material and method sections and or figure legends
Wild animals	The study did not involve wild animals
Field-collected samples	The study did not contain samples collect from the field
Ethics oversight	Animal experiments were approved by the Animal Ethics Committees of the Netherlands Cancer Institute

Note that full information on the approval of the study protocol must also be provided in the manuscript.

Flow Cytometry

Plots

Confirm that:

☒ The axis labels state the marker and fluorochrome used (e.g. CD4-FITC).

☒ The axis scales are clearly visible. Include numbers along axes only for bottom left plot of group (a 'group' is an analysis of identical markers).

☒ All plots are contour plots with outliers or pseudocolor plots.

☒ A numerical value for number of cells or percentage (with statistics) is provided.

Methodology

Sample preparation	Cells were trypsinized and pelleted round bottom FACS tubes. Cell pellets were washed once with Ice cold PBS and incubated with 1:100/1:50 primary antibodies dissolved in 1% BSA in 1x PBS and incubated for 1 hour on ice. In the cases of CD491/b staining the cells were then washed once with 1% BSA in 1x PBS and incubated with secondary antibody (1:400) in 1% BSA in 1x PBS for 1 hour on ice. Pellets were washed once more with 1% BSA in 1x PBS and dissolved in 100 uL 1% BSA in 1x PBS.
Instrument	BD LSR Fortessa Cell Analyzer
Software	The data was collected using BD FACSDiva Software and analysed using FlowJo (BD Inc, USA)
Cell population abundance	No cell sorting was performed
Gating strategy	Based on FSC-A vs SSC-A, cellular debris was excluded (< 50K). Next, FSC-A vs FSC-H was used to exclude doublets and, finally, a staining for propidium iodide was used to exclude dead cells (PI+).

☒ Tick this box to confirm that a figure exemplifying the gating strategy is provided in the Supplementary Information.
